# Promyelocytic Leukemia Protein (PML) Controls *Listeria monocytogenes* Infection

**DOI:** 10.1128/mBio.02179-16

**Published:** 2017-01-10

**Authors:** David Ribet, Valérie Lallemand-Breitenbach, Omar Ferhi, Marie-Anne Nahori, Hugo Varet, Hugues de Thé, Pascale Cossart

**Affiliations:** a Institut Pasteur, Unité des Interactions Bactéries-Cellules, Paris, France; b Inserm, U604, Paris, France; c INRA, USC2020, Paris, France; d Inserm, CNRS, Université Paris Diderot, Institut Universitaire Hématologie, U944/UMR7212, Hôpital St. Louis, Paris, France; e PSL, Collège de France, Paris, France; f Institut Pasteur, Plate-forme Transcriptome et Epigenome, Biomics, Centre d’Innovation et Recherche Technologique (Citech), Paris, France; g Institut Pasteur, Hub Bioinformatique et Biostatistique, Centre de Bioinformatique, Biostatistique et Biologie Intégrative (C3BI, USR 3756 IP CNRS), Paris, France; Johns Hopkins Bloomberg School of Public Health

## Abstract

The promyelocytic leukemia protein (PML) is the main organizer of stress-responsive subnuclear structures called PML nuclear bodies. These structures recruit multiple interactors and modulate their abundance or their posttranslational modifications, notably by the SUMO ubiquitin-like modifiers. The involvement of PML in antiviral responses is well established. In contrast, the role of PML in bacterial infection remains poorly characterized. Here, we show that PML restricts infection by the pathogenic bacterium *Listeria monocytogenes* but not by *Salmonella enterica* serovar Typhimurium. During infection, PML undergoes oxidation-mediated multimerization, associates with the nuclear matrix, and becomes de-SUMOylated due to the pore-forming activity of the *Listeria* toxin listeriolysin O (LLO). These events trigger an antibacterial response that is not observed during *in vitro* infection by an LLO-defective *Listeria* mutant, but which can be phenocopied by specific induction of PML de-SUMOylation. Using transcriptomic and proteomic microarrays, we also characterized a network of immunity genes and cytokines, which are regulated by PML in response to *Listeria* infection but independently from the listeriolysin O toxin. Our study thus highlights two mechanistically distinct complementary roles of PML in host responses against bacterial infection.

## IMPORTANCE

The promyelocytic leukemia protein (PML) is a eukaryotic protein that can polymerize in discrete nuclear assemblies known as PML nuclear bodies (NBs) and plays essential roles in many different cellular processes. Key to its function, PML can be posttranslationally modified by SUMO, a ubiquitin-like modifier. Identification of the role of PML in antiviral defenses has been deeply documented. In contrast, the role of PML in antibacterial defenses remains elusive. Here, we identify two mechanistically distinct complementary roles of PML in antibacterial responses against pathogens such as *Listeria*: (i) we show that PML regulates the expression of immunity genes in response to bacterial infection, and (ii) we unveil the fact that modification of PML SUMOylation by bacterial pore-forming toxins is sensed as a danger signal, leading to a restriction of bacterial intracellular multiplication. Taken together, our data reinforce the concept that intranuclear bodies can dynamically regulate important processes, such as defense against invaders.

## INTRODUCTION

Promyelocytic leukemia protein (PML) is a protein originally identified as part of a t(15:17) chromosomal translocation resulting in the fusion of *PML* and retinoic acid receptor alpha genes in acute promyelocytic leukemia (APL) patients (1–5). In normal cells, PML is present both as a diffuse form in the nucleoplasm and cytoplasm and polymerized in discrete subnuclear structures known as PML nuclear bodies (NBs). PML proteins define the boundaries of these NBs, which constitute non-membrane-bound compartments in the nucleoplasm (6–8). PML NBs are dynamic stress-responsive structures that constitutively or transiently have the ability to recruit a large number of proteins. Studies investigating the basis for arsenic trioxide-initiated APL cure have suggested that oxidative stress promotes PML multimerization and PML NB formation (6, 7, 9–12). PML NBs may also regulate the posttranslational modifications of recruited proteins, thereby controlling their sequestration, activation, or stability (11–13). In agreement with its large and diverse repertoire of interacting partners, PML is involved in many different cellular processes, such as senescence, apoptosis, or antiviral defense (6, 7, 14–17). The role of PML in antiviral defense is illustrated by the higher sensitivity of PML knockout mice to different viruses (reviewed in reference 16). Many viruses counteract this PML antiviral activity by decreasing PML expression or stability or by altering PML NB integrity (16, 17).

*Listeria monocytogenes* is a Gram-positive bacterium that is responsible for the foodborne disease listeriosis. Although well adapted to survive extracellularly, this pathogen can also infect, survive, and replicate in the cytoplasm of both macrophages and nonprofessional phagocytic cells, such as epithelial cells (18). The numerous strategies employed by *Listeria* to interfere with host processes have raised this bacterium as one of the best model organisms for the study of bacterial pathogenesis and pathophysiology. Among the different cellular pathways subverted by *Listeria*, we have previously demonstrated that infection of cells by this bacterium was associated with an alteration of the host SUMOylome, i.e., the repertoire of proteins posttranslationally modified by the ubiquitin-like SUMO polypeptide (19, 20). Strikingly, pore formation at the level of the host plasma membrane by listeriolysin O (LLO), a toxin secreted by *Listeria*, triggers the degradation of Ubc9, the unique E2 enzyme of the SUMOylation machinery in humans (20). This degradation leads to an inhibition of *de novo* SUMOylations. De-SUMOylation reactions, catalyzed by the different SUMO isopeptidases of the host cell, then result in a rapid loss of SUMO conjugates. Several nuclear factors, including transcription factors, are de-SUMOylated in response to infection, explaining how host SUMOylome alteration during *Listeria* infection leads to host transcription modifications (21).

Interestingly, other bacterial pathogens were shown to manipulate host SUMOylation machinery during infection. Infection of HeLa cells with *Shigella flexneri*, a pathogen causing bacillary dysentery, leads to a decrease in Ubc9 level and a modification of host SUMOylated proteins (22, 23). Transcription factors involved in inflammatory responses, such as c-FOS, RXRα, and PPAR γ, for example, are de-SUMOylated in response to *Shigella* infection (23). In addition, SUMOylation was reported to restrain production of inflammatory cytokines by silencing *ifnb1* expression (24). Alteration of SUMOylation may thus contribute to the inflammatory response associated with *Shigella*. *Salmonella enterica* serovar Typhimurium, a bacterium responsible for gastroenteritis in humans, was also shown to decrease the Ubc9 level during infection and to alter the host SUMOylome during infection (25). Together, these studies unveiled the role of SUMOylation in the regulation of key host factors controlling infection by different classes of pathogens.

Interestingly, SUMOylation plays a critical role in the function of PML and PML NBs. PML can indeed be SUMOylated on several lysine residues, and PML SUMOylation is required for the recruitment of PML NB partners (26–33). In addition, most PML partners can be SUMOylated, and PML NBs are thought to facilitate this process through the recruitment of Ubc9 upon stress (11). Some of the PML partners are then degraded by SUMO-dependent ubiquitin ligases, such as RNF4 (34, 35). Thus, NBs couple stress to enhanced SUMOylation of PML interactors, enforcing multiple responses, such as TP53 activation, senescence, or antiviral effects (11, 13, 36).

In contrast to PML’s established action against viruses, a single study mentioned that PML knockout mice are more sensitive to infection by *Listeria monocytogenes* (37). However, the exact role of PML in anti-*Listeria* responses or in other bacterial infections has not been elucidated. In this study, we demonstrate that PML restricts *Listeria* infection both *in vitro* and *in vivo*. We identify in particular that PML upregulates several genes and cytokines involved in innate immunity. Moreover, in response to LLO-producing *Listeria*, PML multimerizes, associates with the nuclear matrix, and becomes de-SUMOylated, which ultimately impairs *Listeria* replication. Taken together, our data highlight different roles of PML in antibacterial responses, notably the role for PML’s SUMOylation status in the sensing of and defense against bacterial pathogens that produce pore-forming toxins. Our findings further illustrate the concept that intranuclear bodies dynamically regulate important processes, such as defense against invaders.

## RESULTS

### Depletion of PML increases *Listeria* infection.

**FIG 1:**
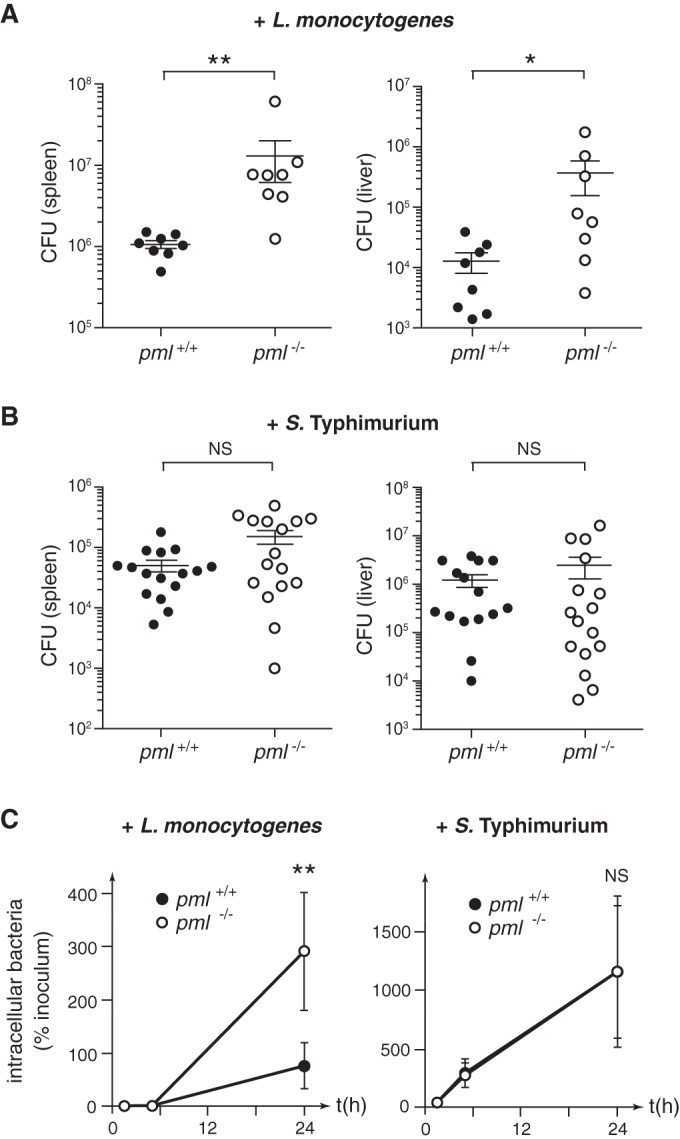
PML restricts *Listeria* infection. (A and B) *pml*^+/+^ and *pml*^−/−^ mice were infected with *L. monocytogenes* (A) or *S*. Typhimurium (B), and the numbers of CFUs per spleen and liver were quantified 72 h after infection (bars and whiskers show mean results ± standard errors of the means [SEM]; *, *P* < 0.05; **, *P* < 0.01; NS, not significant; Mann-Whitney statistical test). (C) *pml*^+/+^ or *pml*^−/−^ MEFs were infected with *L. monocytogenes* or *S*. Typhimurium, and the numbers of intracellular bacteria, represented as the percentages of the inoculum used for infection, were quantified (mean results ± SEM from 6 to 8 independent experiments; **, *P* < 0.01; NS, not significant; unpaired two-tailed Student’s *t* test).

In order to characterize the role of PML during infection *in vivo*, we first infected wild-type (WT) and PML knockout mice with *Listeria* (EGD strain). As mice are poorly permissive for oral infections with *Listeria*, due to lack of recognition of murine E-cadherin by the essential *Listeria* internalin A surface protein (InlA) (38), we used the intravenous route to perform infections. We enumerated CFUs (colony-forming units) in the liver and spleen of animals 3 days after infection (Fig. 1A). We observed a significantly higher number of CFUs per organ in *pml*^−/−^ mice than in *pml*^+/+^ mice, confirming that *pml*^−/−^ mice are more sensitive to *Listeria* infection (37). We then similarly challenged *pml*^+/+^ and *pml*^−/−^ mice with *Salmonella* Typhimurium. In contrast to our observations with *Listeria*, we did not observe a significant difference in the number of CFUs per organ between wild-type and PML-deficient mice, thus revealing a specific defect of *pml*^−/−^ mice in their responses against *Listeria* compared to their responses against another intracellular pathogen (Fig. 1B).

In order to further characterize the role of PML in bacterial infection, we then compared the infection efficiencies of *Listeria* (EGD strain) in immortalized mouse embryonic fibroblasts (MEFs) derived from *pml*^+/+^ or *pml*^−/−^ mice. The numbers of intracellular *Listeria* bacteria were quantified after 1.5 h, 5 h, and 24 h of infection (Fig. 1C). We did not observe significant differences in bacterial numbers after 1.5 h of infection, suggesting that the internalization efficiencies of *Listeria* are similar in *pml*^+/+^ and *pml*^−/−^ MEFs (see Fig. S1A in the supplemental material). In contrast, the number of intracellular bacteria after 24 h of infection was significantly higher in *pml*^−/−^ than in *pml*^+/+^ MEFs (Fig. 1C). These data indicate that *Listeria*’s intracellular replication is facilitated in the absence of PML in MEFs, thus demonstrating that PML restricts the bacterial replication in nonphagocytic cells. In parallel, we infected MEFs with another strain of *Listeria*, EGDe.PrfA* (an EGD-e strain in which PrfA, the master regulator of *Listeria* virulence genes, is constitutively active [39]). A significant increase in the number of intracellular CFUs was again observed in *pml*^−/−^ MEFs compared to the number in *pml*^+/+^ MEFs, confirming the role of PML in the control of *Listeria* infection (see Fig. S1B). Interestingly, infection of MEFs with *Salmonella* did not show significant differences in bacterial numbers at 1.5 h, 5 h, or 24 h, indicating that *pml*^+/+^ and *pml*^−/−^ MEFs are similarly sensitive to *Salmonella* entry and replication and thereby confirming our *in vivo* data and PML’s specificity toward *Listeria* (Fig. 1C; see also Fig. S1A). Together, these data highlight that PML specifically restricts *Listeria* infection both *in vitro* and *in vivo*.

10.1128/mBio.02179-16.1 FIG S1  PML restricts bacterial intracellular replication but not bacterial entry in MEFs. MEFs derived from *pml*^+/+^ or *pml*^−/−^ mice were infected by *L. monocytogenes* or S. Typhimurium. (A) Histograms correspond to the numbers of intracellular bacteria recovered after 1.5 h of infection, expressed as the percentages of the inoculum initially used for infection (mean results ± SEM from 6 to 8 independent experiments; NS, not significant; unpaired two-tailed Student’s *t* test). No significant differences were observed in bacterial numbers, suggesting that the internalization efficiencies of *Listeria* and *Salmonella* are similar in both *pml*^+/+^ and *pml*^−/−^ MEFs. (B) Histograms correspond to fold changes in bacterial intracellular replication, expressed as the ratio of intracellular bacteria at 24 h versus 1.5 h of infection (mean results ± SD from 3 independent experiments; **, *P* < 0.01; unpaired two-tailed Student’s *t* test). A significant increase in the replication efficiency is observed in *pml*^−/−^ MEFs compared to that of *pml*^+/+^ MEFs, indicating that PML restricts the intracellular replication of *Listeria* strain EGDe.PrfA*. Download FIG S1, PDF file, 1.6 MB. Copyright © 2017 Ribet et al. 2017 Ribet et al. This content is distributed under the terms of the Creative Commons Attribution 4.0 International license.

### PML regulates the expression of genes involved in innate immunity.

**FIG 2:**
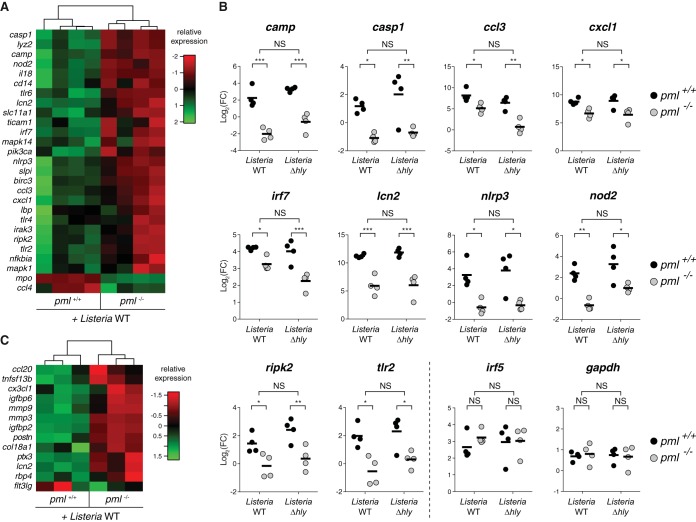
PML regulates gene expression and cytokine production. (A) Heat map of the 27 genes identified as significantly differentially regulated in *pml*^+/+^ versus *pml*^−/−^ MEFs after 24 h of infection with wild-type *Listeria* (Benjamini and Hochberg method, adjusted *P* value of <0.05; data from 4 independent experiments). Color code indicates relative expression compared with the expression in uninfected MEFs. (B) Expression levels of 10 representative genes differentially regulated in *pml*^+/+^ versus *pml*^−/−^ MEFs after infection with either wild-type or Δ*hly Listeria*. Data represent fold changes (FC) of gene expression levels in infected compared to uninfected MEFs (bars show mean results from 4 independent experiments; *, *P* < 0.05; **, *P* < 0.01; ***, *P* < 0.001; NS, not significant). *irf5* and *gapdh* are shown as examples of genes not regulated by PML. (C) Heat map of the 13 cytokines identified as significantly differentially secreted by *pml*^+/+^ versus *pml*^−/−^ MEFs after 24 h of infection with wild-type *Listeria* (Benjamini and Hochberg method, adjusted *P* value of <0.05; data from 3 independent experiments). Color code indicates relative expression compared with the expression in uninfected MEFs.

To better characterize the role of PML during bacterial infection, we compared gene expression and cytokine production in *pml*^+/+^ and *pml*^−/−^ MEFs after 24 h of infection with *Listeria* (EGD strain). RNAs extracted from infected MEFs were analyzed to profile the expression of 84 genes involved in the innate immune response using transcriptomic microarrays. We could identify 27 genes, including *camp*, *casp1*, *ccl3*, *cxcl1*, *irf7*, *lcn2*, *nlrp3*, *nod2*, *ripk2*, and *tlr2*, that were differentially expressed in *pml*^+/+^ versus *pml*^−/−^ MEFs following infection (Fig. 2; see also Table S1A in the supplemental material). This indicates that PML plays an important role in the regulation of these genes. We conducted a similar approach to identify putative PML-regulated cytokines. We collected supernatants from control and 24-h-infected *pml*^+/+^ and *pml*^−/−^ MEFs and monitored the presence of 110 different cytokines using proteome microarrays. Again, we could identify 13 cytokines that were expressed in a PML-dependent manner (Fig. 2; see also Table S2A). Among these PML-regulated cytokines, we identified in particular *ccl20* and *cx3cl1*, 2 cytokines known to play key roles in antibacterial responses.

10.1128/mBio.02179-16.6 Table S1  Relative gene expression levels in *pml*^−/−^ versus *pml*^+/+^ MEFs after infection with *Listeria*. Gene expression was determined by quantitative PCR (qPCR) analysis of RNAs from *pml*^−/−^ and *pml*^+/+^ MEFs infected for 24 h with wild-type *Listeria* (A) or a Δ*hly Listeria* mutant (B). Data represent fold changes of gene expression levels in *pml*^−/−^ MEFs compared to the levels in *pml*^+/+^ MEFs (mean results from 4 independent experiments). Genes that had fold changes with an adjusted *P* value of <0.05 (Benjamini and Hochberg method) are considered differentially regulated between the two conditions being compared. Download Table S1, XLSX file, 0.1 MB. Copyright © 2017 Ribet et al. 2017 Ribet et al. This content is distributed under the terms of the Creative Commons Attribution 4.0 International license.

10.1128/mBio.02179-16.7 Table S2  Relative cytokine production levels in *pml*^−/−^ versus *pml*^+/+^ MEFs after infection with *Listeria*. Cytokine production was quantified using supernatants from *pml*^−/−^ and *pml*^+/+^ MEFs infected for 24 h with wild-type *Listeria* (A) or a Δ*hly Listeria* mutant (B). Data represent fold changes of cytokine production levels in *pml*^−/−^ MEFs compared to the levels in *pml*^+/+^ MEFs (mean results from 3 independent experiments). Cytokines that had fold changes with an adjusted *P* value of <0.05 (Benjamini and Hochberg method) are considered differentially produced between the two conditions being compared. Download Table S2, XLSX file, 0.1 MB. Copyright © 2017 Ribet et al. 2017 Ribet et al. This content is distributed under the terms of the Creative Commons Attribution 4.0 International license.

As LLO was shown to be a major determinant of host response to infection (40), we tested whether some of these PML-regulated genes or cytokines were expressed in an LLO-dependent manner. To do so, we analyzed *pml*^+/+^ and *pml*^−/−^ MEFs infected with an LLO-defective *Listeria* mutant (*Listeria* Δ*hly*). We observed that all genes and cytokines differentially regulated by PML in cells infected with wild-type bacteria were also differentially regulated in response to the Δ*hly Listeria* mutant (Fig. 2; see also Tables S1B and S2B). Thus, the PML-regulated genes and cytokines identified here are expressed in an LLO-independent manner. We finally monitored gene expression, using the same transcriptomic microarrays, in *pml*^+/+^ MEFs treated or not with purified LLO. No gene from the 27 targets identified as being regulated by PML during wild-type *Listeria* infection was differentially expressed after LLO treatment (see Table S3). This confirms our results showing that PML-dependent genes modulated in response to *Listeria* infection are independent of LLO.

10.1128/mBio.02179-16.8 Table S3  Relative gene expression levels in *pml*^+/+^ MEFs treated or not with LLO. Gene expression was determined by qPCR analysis of RNAs from *pml*^+/+^ MEFs treated or not with 3 nM LLO for 30 min. Data represent fold changes of gene expression levels in LLO-treated versus untreated *pml*^+/+^ MEFs (mean results from 3 independent experiments). Genes that had fold changes with an adjusted *P* value of >0.05 (Benjamini and Hochberg method) are considered not differentially regulated between the two conditions being compared. Download Table S3, XLSX file, 0.1 MB. Copyright © 2017 Ribet et al. 2017 Ribet et al. This content is distributed under the terms of the Creative Commons Attribution 4.0 International license.

In conclusion, our transcriptomic and proteomic screens identified a network of genes induced upon infection in a PML-dependent manner and highlighted a first role of PML in antibacterial responses, acting as a master regulator of genes involved in immunity against *Listeria*.

### PML restricts *Listeria* replication in an LLO-dependent manner.

**FIG 3:**
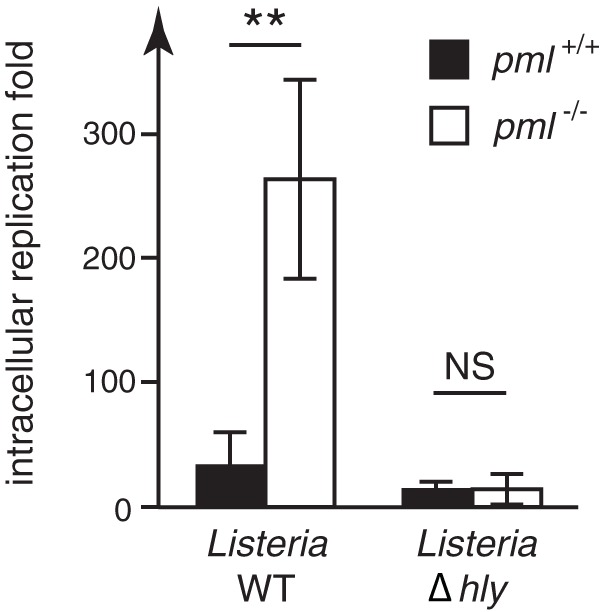
PML impairs replication of LLO-producing *Listeria*. *pml*^+/+^ and *pml*^−/−^ MEFs were infected with wild-type and Δ*hly Listeria* strains. The fold changes in intracellular replication were determined by calculating the ratio of intracellular bacteria at 24 h versus 1.5 h of infection (mean results ± standard deviations [SD] from 3 independent experiments; **, *P* < 0.01; NS, not significant; unpaired two-tailed Student’s *t* test).

To get further insights into how PML may restrict *Listeria*’s intracellular replication, we compared the replication efficiencies of wild-type and Δ*hly Listeria* in *pml*^+/+^ or *pml*^−/−^ MEFs. We took advantage of the observation that in our MEFs, and in contrast to other murine cell lines (41), a fraction of Δ*hly* bacteria can escape from the internalization vacuole and replicate intracellularly (Fig. 3; see also Fig. S2 in the supplemental material). MEFs were infected with *Listeria* and the numbers of intracellular bacteria were quantified after 1.5 h and 24 h of infection. Strikingly, we observed that, in contrast to the parental wild-type *Listeria* strain, the replication efficiencies of the Δ*hly* mutant were similar in *pml*^+/+^ and *pml*^−/−^ MEFs (Fig. 3). This result demonstrates that PML restricts only LLO-producing bacteria and suggests that LLO exposure triggers, via PML, a host response against *Listeria*.

10.1128/mBio.02179-16.2 FIG S2  Detection of *Listeria* vacuolar escape in MEFs. Immunofluorescence analysis of *pml*^+/+^ MEFs transfected with an expression vector for YFP-CBD, a YFP chimera protein of the cell wall binding domain from the *Listeria* phage endolysin Ply118 (R. Henry, L. Shaughnessy, M. J. Loessner, C. Alberti-Segui, D. E. Higgins, and J. A. Swanson, Cell Microbiol 8:107–119, 2006, doi: 10.1111/j.1462-5822.2005.00604.x). MEFs were infected 24 h after transfection with wild-type or Δ*hly Listeria monocytogenes*. After 1 h of infection, cells were washed and incubated for an additional 4 h in culture medium supplemented with gentamicin to kill extracellular bacteria; intracellular bacteria remain unaffected by this antibiotic. Cells were then fixed, and bacterial and cellular DNAs were stained with 4′,6-diamidino-2-phenylindole (DAPI) (scale bar, 5 μm). The YFP-CBD protein, synthesized in the host cell cytoplasm, recognizes and binds only to bacteria that escaped from the internalization vacuole. Here, CBD-positive Δ*hly Listeria* cells can be detected, indicating that at least a fraction of these bacteria can escape the internalization vacuole in this murine cell line and reach the host cell cytosol, as already observed for several human cell lines (M. A. Hamon, D. Ribet, F. Stavru, and P. Cossart, Trends Microbiol 20:360–368, 2012, doi: 10.1016/j.tim.2012.04.006). Download FIG S2, PDF file, 3.4 MB. Copyright © 2017 Ribet et al. 2017 Ribet et al. This content is distributed under the terms of the Creative Commons Attribution 4.0 International license.

As the results for the PML-regulated genes and cytokines identified above were similar in cells infected with wild-type and Δ*hly Listeria*, we hypothesized that PML restricts LLO-producing bacteria by an additional, LLO-triggered pathway.

### LLO triggers PML de-SUMOylation.

**FIG 4:**
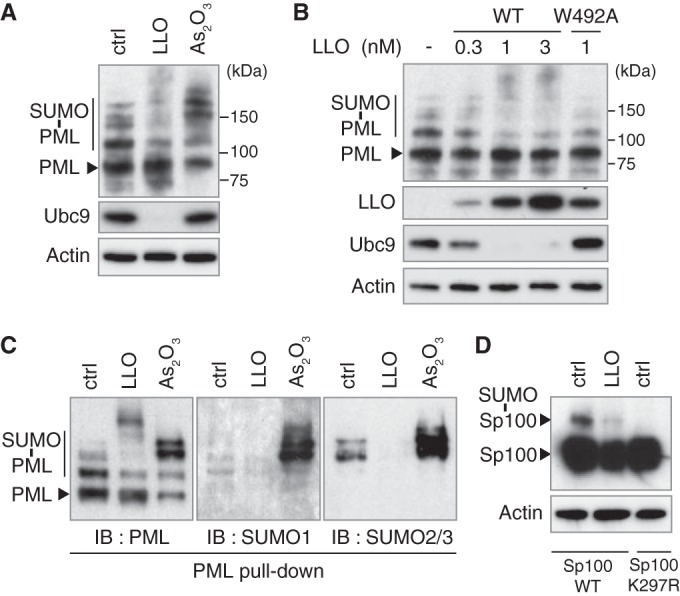
LLO induces PML and Sp100 de-SUMOylation. (A and B) Immunoblot analysis, using anti-PML, anti-Ubc9, anti-actin, and anti-LLO antibodies, of whole-cell lysates from CHO-PML cells treated with 1 nM LLO for 20 min or 10 μM As_2_O_3_ for 1 h (A) or treated for 20 min with either increasing doses of WT LLO or the non-pore-forming LLO^W492A^ mutant (B). (C) Immunoblot analysis, using anti-PML, anti-SUMO1 and anti-SUMO2/3 antibodies, of PML proteins from CHO-PML cells treated with 1 nM LLO for 20 min or with 10 μM As_2_O_3_ for 1 h; the PML proteins were pulled down by means of His_6_ tags. (D) Immunoblot analysis, using anti-Sp100 and anti-actin antibodies, of whole-cell lysates from HeLa cells transfected with expression vectors for wild-type Sp100 or a non-SUMOylatable Sp100^K297R^ mutant and treated with 3 nM LLO for 30 min. All immunoblots displayed were done under reducing conditions (+DTT).

In order to explore the link between LLO-producing *Listeria* and PML, we assessed whether infection leads to a modification of PML SUMOylation. We had previously demonstrated that pore formation in the host plasma membrane by the *Listeria* toxin LLO triggers de-SUMOylation of several host proteins during infection (20, 21). We treated CHO (Chinese hamster ovary) cells stably expressing a His_6_-tagged version of the human PML protein (isoform III [PML-III]) with sublytic concentrations of purified LLO (from 0.3 to 3 nM). As a control, we treated CHO-PML cells in parallel with arsenic trioxide (As_2_O_3_), a drug successfully used to treat APL, which promotes PML multimerization and SUMOylation through Ubc9 recruitment to PML NBs (9, 30, 42, 43; reviewed in reference 44). Using immunoblot analysis of whole-cell extracts, we showed that, in sharp contrast to the effect of As_2_O_3_, which rapidly increases PML SUMOylation, LLO triggers a dose-dependent decrease in the level of PML high-molecular-weight species, corresponding to the well-described SUMOylated forms of PML (Fig. 4A and B). Of note, the total level of PML protein is not affected by LLO, pointing to de-SUMOylation events rather than degradation of PML, which is likely caused by the concomitant LLO-mediated degradation of Ubc9 (Fig. 4A and B). PML is thus a target of LLO-triggered loss of SUMOylation.

We further assessed that the effect of LLO on PML SUMOylation is pore dependent by using an LLO mutant (with a W-to-A change at position 492 [LLO^W492A^]) that is able to bind cellular membranes but unable to form pores (20). This mutant did not affect PML SUMOylation or the Ubc9 level, in line with our previous findings that de-SUMOylation events mediated by LLO are pore dependent (Fig. 4B).

To confirm the effect of LLO on PML SUMOylation, we pulled down His_6_-tagged PML proteins from CHO-PML cells treated with LLO or As_2_O_3_. Immunoblot analysis of the pulled-down PML reveals an increase in the intensity of PML SUMOylated forms after As_2_O_3_ treatment, whereas LLO decreases PML SUMOylation, particularly by the SUMO2/3 paralog (Fig. 4C). LLO can induce the degradation of some host SUMOylated proteins (20). To rule out a possible proteasome-dependent degradation of PML SUMOylated forms in response to LLO, we pretreated cells with the proteasome inhibitor MG132 and showed that this treatment does not block the LLO-induced decrease in PML SUMOylated forms (see Fig. S3 in the supplemental material). Together, our data show that the plasma membrane pores formed by LLO lead to PML de-SUMOylation but not degradation. This is in sharp contrast to the effect of As_2_O_3_, which triggers PML hyper-SUMOylation, followed by PML polyubiquitylation and proteasomal degradation (30, 34, 35).

10.1128/mBio.02179-16.3 FIG S3  LLO induces PML de-SUMOylation rather than degradation. (A) Immunoblot analysis using anti-K48-linked polyubiquitin and anti-actin antibodies of whole-cell lysates from CHO-PML cells preincubated with 10 μM MG132 for 5 h. The level of proteins conjugated to K48-polyubiquitin chains increased after MG132 treatment, thus validating proteasome inhibition under these conditions. (B) Immunoblot analysis using anti-PML, anti-Ubc9, and anti-actin antibodies of whole-cell lysates from CHO-PML cells preincubated with 10 μM MG132 for 5 h and then treated with 1 nM LLO for 20 min. Pretreatment with MG132 does not block loss of PML SUMOylated forms, suggesting that LLO triggers PML de-SUMOylation rather than degradation. Download FIG S3, PDF file, 1.5 MB. Copyright © 2017 Ribet et al. 2017 Ribet et al. This content is distributed under the terms of the Creative Commons Attribution 4.0 International license.

We finally monitored whether LLO also affects the SUMOylation level of Sp100, a constitutive structural component of PML NBs (45). HeLa cells transfected with expression vectors for wild-type Sp100 or a non-SUMOylatable Sp100^K297R^ mutant were treated with LLO. Immunoblot analysis of whole-cell lysates shows a strong decrease in the level of the SUMO-modified form of Sp100 in response to LLO, indicating that this toxin triggers de-SUMOylation of not only PML but also other essential PML NB components (Fig. 4D).

### LLO triggers PML multimerization.

**FIG 5:**
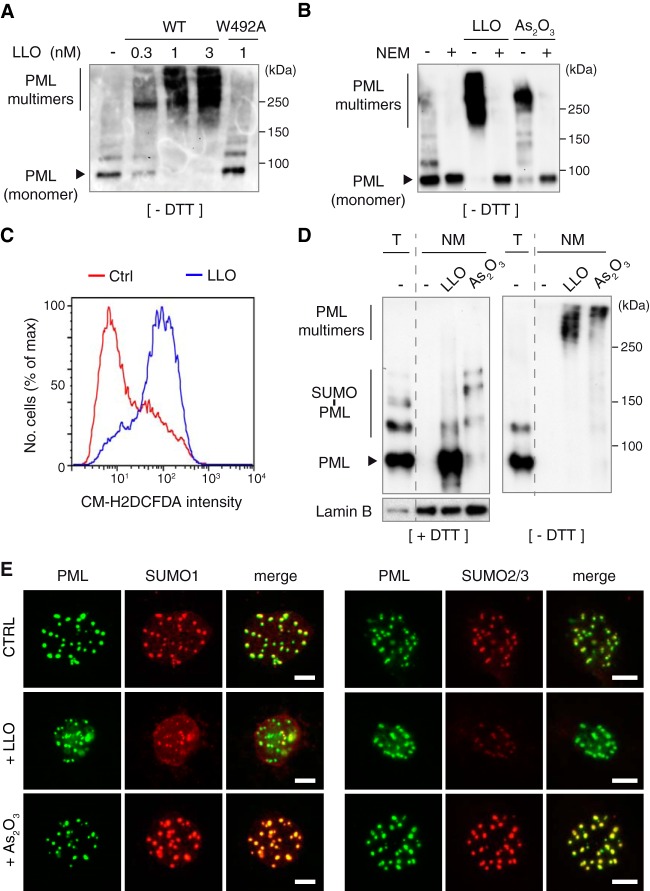
LLO induces PML multimerization and association with the nuclear matrix. (A and B) Immunoblot analysis under nonreducing conditions (without DTT [-DTT]), using anti-PML antibodies, of whole-cell lysates from CHO-PML cells. Cells were treated for 20 min with different doses of wild-type LLO or with LLO^W492A^ mutant (A) or pretreated with 100 μM NEM for 1 h and then treated with 1 nM LLO for 20 min or with 10 μM As_2_O_3_ for 1 h (B). (C) Representative results of flow cytometry analysis of CHO cells labeled with the ROS-sensitive CM-H_2_DCFDA probe after incubation with 0.3 nM LLO for 2 min. (D) Immunoblot analysis under reducing (+DTT) or nonreducing (-DTT) conditions, using anti-PML and anti-lamin B antibodies, of total cell lysates (T) or nuclear matrix preparations (NM) from CHO-PML cells treated with 1 nM LLO for 20 min or 10 μM As_2_O_3_ for 1 h. (E) Immunofluorescence analysis, using anti-PML, anti-SUMO1, and anti-SUMO2/3 antibodies, of nuclear matrices from CHO-PML cells treated with 1 nM LLO for 20 min or 10 μM As_2_O_3_ for 1 h. Scale bar, 5 μm.

Short treatments with oxidative agents like As_2_O_3_ promote PML NB formation by inducing PML multimerization and association with the nuclear matrix, a nuclear fraction characterized by its insolubility and resistance to high salt and nuclease extractions (9, 11, 27, 30, 43, 46). LLO, like other pore-forming toxins (47), rapidly induces the production of reactive oxygen species (ROS) in the host cell (Fig. 5). We thus tested whether LLO would trigger PML multimerization. We performed immunoblot analysis of whole-cell lysates obtained under nonreducing conditions from LLO-treated CHO-PML cells. We observed PML multimers (with molecular masses above 200 kDa) that were not detected either under reducing conditions (Fig. 5A and Fig. 4) or when cells were pretreated with *N*-ethylmaleimide (NEM), an alkylating reagent that blocks free thiols (Fig. 5B). Altogether, these results strongly suggest that LLO induces an oxidative stress that leads to PML multimerization via intermolecular disulfide bonds, as observed for As_2_O_3_ (9, 11).

To decipher whether LLO triggers an association of PML with the nuclear matrix similarly to As_2_O_3_, we performed *in situ* high-salt extraction and DNase/RNase treatments to isolate nuclear matrices from LLO- or As_2_O_3_-treated CHO-PML cells. We observed that LLO-induced PML multimers were strongly associated with the nuclear matrix (Fig. 5D). Interestingly, the predominant forms of nuclear matrix-associated PML are highly SUMOylated in As_2_O_3_-treated cells, as previously observed (9), but are poorly SUMOylated in LLO-treated cells (Fig. 5D). Finally, we performed immunofluorescence analysis of nuclear matrices obtained from CHO-PML cells treated with LLO or As_2_O_3_. Staining with anti-PML antibodies showed that LLO does not disrupt PML NBs (Fig. 5E). Staining with anti-SUMO1 and anti-SUMO2/3 antibodies revealed that LLO induces decreases of both SUMO1 and SUMO2/3 labeling of nuclear matrix-associated-PML, in sharp contrast to As_2_O_3_, which increases SUMO1 and SUMO2/3 labeling of these structures (Fig. 5E; see also Fig. S4 in the supplemental material).

10.1128/mBio.02179-16.4 FIG S4  Control of *in situ* nuclear matrix preparations. Immunofluorescence analysis of total nuclei from CHO-PML cells or nuclear matrix preparations using DAPI, anti-PML, anti-lamin B, and anti-RXRα antibodies. As expected, after the high-salt extraction and nuclease treatment used for nuclear matrix preparation, the nuclear soluble RXRα factor and DNA were removed, whereas lamin B and NB-associated PML, which belong to the nuclear matrix fraction, are still present. Scale bar, 5 μm. Download FIG S4, PDF file, 3.4 MB. Copyright © 2017 Ribet et al. 2017 Ribet et al. This content is distributed under the terms of the Creative Commons Attribution 4.0 International license.

Taken together, our results show that exposure of host cells to LLO induces a covalent multimerization of PML proteins and their association with the nuclear matrix. However, in contrast to As_2_O_3_, exposure to LLO leads to a strong decrease in PML SUMOylation, consistent with the loss, rather than NB recruitment, of Ubc9.

### Other bacterial pore-forming toxins trigger PML de-SUMOylation and multimerization.

**FIG 6:**
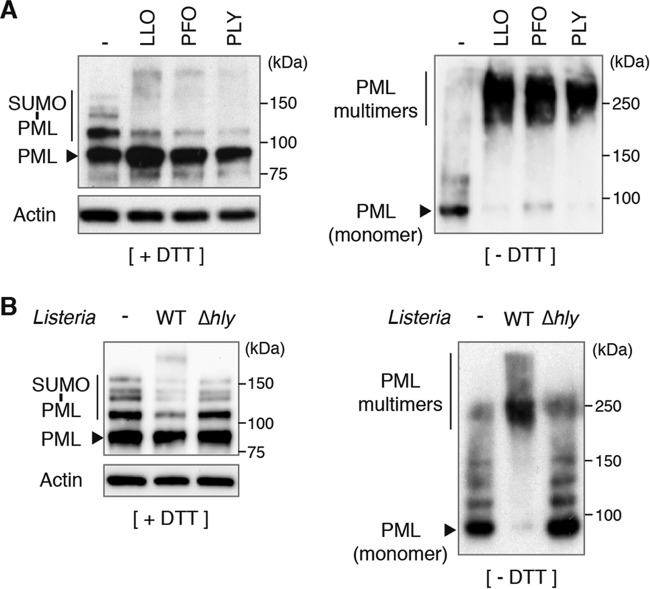
PML multimerizes and is de-SUMOylated in response to different pore-forming toxins and to *Listeria* infection. (A and B) Immunoblot analysis under reducing (+DTT) or nonreducing (-DTT) conditions, using anti-PML and anti-actin antibodies, of whole-cell lysates from CHO-PML cells treated for 20 min with different pore-forming toxins (A) or infected for 5 h with wild-type or Δ*hly Listeria* (B).

The degradation of Ubc9 and de-SUMOylation of host proteins can be triggered by other bacterial pore-forming toxins belonging, like LLO, to the family of cholesterol-dependent cytolysins (20, 48). We thus treated CHO-PML cells with two of these toxins, perfringolysin O (PFO; from *Clostridium perfringens*) and pneumolysin (PLY; from *Streptococcus pneumoniae*), and showed that these pore-forming toxins also lead to PML de-SUMOylation and multimerization (Fig. 6A). Thus, PML modifications may occur in response to different bacteria that produce pore-forming toxins.

### Infection by *Listeria* affects PML SUMOylation and multimerization.

In order to assess whether the PML modifications observed in response to purified LLO are also induced in the context of bacterial infection, we infected CHO-PML cells with wild-type or Δ*hly Listeria*. Cells were lysed after 5 h of infection and analyzed by immunoblotting experiments. We observed that infection with wild-type *L. monocytogenes* induces a strong multimerization of PML, associated with its global de-SUMOylation (Fig. 6B). Modifications of PML were not observed during infection with the Δ*hly Listeria* mutant (Fig. 6B). These results demonstrate that *Listeria* infection triggers PML multimerization and de-SUMOylation in an LLO-dependent manner.

### LLO-induced PML de-SUMOylation impairs *Listeria*’s intracellular replication.

**FIG 7:**
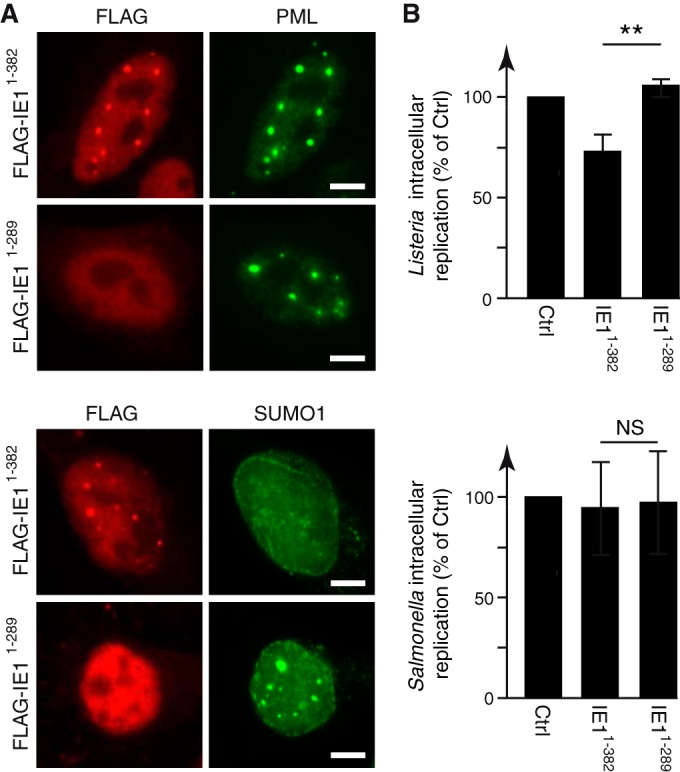
Induction of PML de-SUMOylation impairs *Listeria*’s intracellular replication. (A) Immunofluorescence analysis of nuclei from HeLa cells transfected with expression vectors for FLAG-iE1^1–382^ or FLAG-iE1^1–289^ and stained with anti-FLAG, anti-PML, and anti-SUMO1 antibodies. Scale bar, 5 μm. (B) HeLa cells transfected with FLAG-iE1^1–382^ or FLAG-iE1^1–289^ were infected with *Listeria* or *Salmonella*. Fold changes in intracellular replication were calculated as the ratio of intracellular bacteria at 24 h versus 1.5 h of infection and are expressed as percentages of the result for the control (mean results ± SD from 3 to 4 independent experiments; **, *P* < 0.01; NS, not significant; unpaired two-tailed Student’s *t* test).

Our results suggest that LLO-induced PML de-SUMOylation may trigger host antibacterial responses, impairing bacterial replication. To directly explore the role of PML de-SUMOylation in *Listeria*’s intracellular replication, we transfected HeLa cells with a vector expressing a truncated form of the iE1 protein from human cytomegalovirus (including residues 1 to 382 [iE1^1–382^]). This protein induces PML de-SUMOylation without disrupting PML NBs, although other effects on PML cannot be formally excluded (Fig. 7A; see also Fig. S5 in the supplemental material) (49). We used as controls cells transfected with an empty vector or with an expression vector for a shorter iE1 protein (iE1^1–289^) that does not trigger PML de-SUMOylation (Fig. 7A; see also Fig. S5). HeLa cells transfected with these different plasmids were then infected by *Listeria*, and the numbers of intracellular bacteria were quantified 1.5 h and 24 h after infection (Fig. 7B). Strikingly, we observed a consistent decrease in the replication efficiency of bacteria in cells transfected with iE1^1–382^ compared to the bacterial replication in cells transfected with an empty vector or with iE1^1–289^ (Fig. 7B). In contrast, when the same experiment was performed with *Salmonella*, similar intracellular replication efficiencies were observed under all test conditions, indicating that PML SUMOylation does not restrict this bacterium, in agreement with our previous *in vivo* and *in vitro* data (Fig. 1B and C and 7B). Taken together, these data establish that PML de-SUMOylation impairs *Listeria* infection in the host cell, thereby supporting our hypothesis that LLO-induced PML SUMO deconjugation is a contributor to bacterial replication dampening.

10.1128/mBio.02179-16.5 FIG S5  hCMV iE1^1–382^ protein binds to PML and induces its de-SUMOylation. (A) Immunoblot analysis using anti-FLAG and anti-actin antibodies of HeLa cells transfected with pCDNA.3 empty vector or expression vectors for FLAG-iE1^1–382^ or iE1^1–289^. (B) Immunofluorescence analysis of nuclei from transfected HeLa cells stained with DAPI, anti-FLAG, anti-PML, and anti-SUMO1 antibodies. The iE1^1–382^ protein displays a nuclear localization, binds to PML, and induces its de-SUMOylation. The iE1^1–289^ protein also has a nuclear localization but does not bind or de-SUMOylate PML. Scale bar, 5 μm. Download FIG S5, PDF file, 3.9 MB. Copyright © 2017 Ribet et al. 2017 Ribet et al. This content is distributed under the terms of the Creative Commons Attribution 4.0 International license.

## DISCUSSION

**FIG 8:**
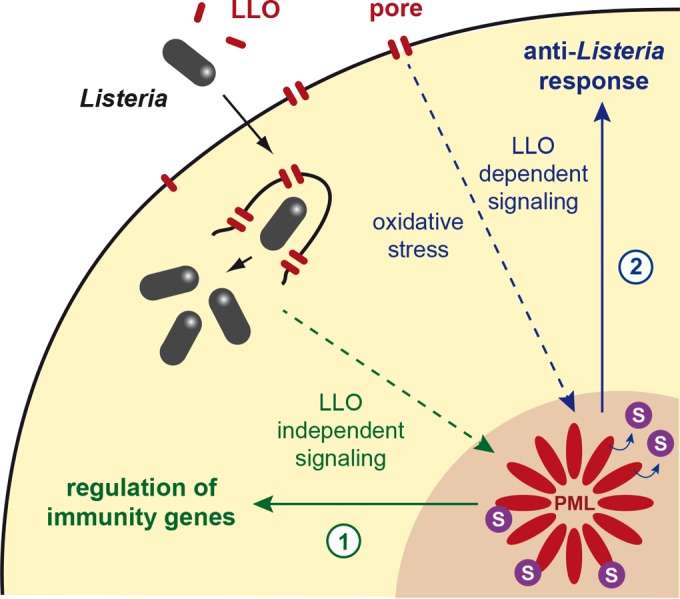
Model illustrating the roles of PML and its SUMOylation in anti-*Listeria* responses. (1) Infection with *Listeria* triggers the expression of genes involved in innate immunity. A fraction of these genes are regulated by PML, in an LLO-independent manner. (2) Pore formation in the host plasma membranes by LLO induces Ubc9 degradation and oxidative stress, leading to PML de-SUMOylation, multimerization, and association with the nuclear matrix. We propose that this state of PML is sensed as a danger signal by the cell, which triggers in response the dampening of bacterial replication.

In this study, we analyzed the role of PML and its SUMOylation in bacterial infection. By studying MEFs’ responses to *Listeria* infection, we characterized a network of genes and cytokines that are involved in innate immunity and regulated by PML. The lack of induction of these genes in *pml*^−/−^ cells may explain, albeit partially, the increased sensitivity of these mice to *Listeria* infection. Of note, we established that the expression of these genes does not depend on the presence of LLO (Fig. 8). Previous studies have established that PML regulates either positively or negatively the expression of genes involved in antiviral responses and, more particularly, of interferon (IFN)-stimulated genes (ISGs) (50–55). Here, in the context of *Listeria* infection, 17 of the 36 PML-regulated genes and cytokines identified are actually known IFN-inducible genes (based on the Interferome database, version 2.01 [56]). This suggests that some of the mechanisms involved in the PML regulation of ISGs in the context of viral infection might be shared during infection by a bacterial pathogen, such as *Listeria*. Besides IFN-inducible genes, PML was also shown to regulate NF-kB-dependent genes, such as interleukin-6 (IL-6) (37, 53). Here, in the context of *Listeria* infection, we do not observe a significant difference between *pml*^+/+^ and *pml*^−/−^ MEFs in their expression of *il6* or other established NF-kB-dependent genes, such as *tnf-*α (encoding tumor necrosis factor alpha), *ccl5*, *il1b* (encoding interleukin-1b), *il12a*, *il12b*, and *trl9* (see Tables S1 and S2 in the supplemental material). This suggests that PML does not regulate NF-kB genes in the context of *Listeria* infection.

In addition to this role of PML in the regulation of immunity genes, we demonstrate that, in MEFs, PML restricts specifically the replication of LLO-expressing *Listeria* but not that of a Δ*hly* mutant. As PML-dependent genes were similarly regulated in cells infected with wild-type and Δ*hly Listeria*, we conclude that PML restricts *Listeria* by an additional, LLO-dependent mechanism, relying, at least in part, on PML de-SUMOylation (Fig. 8). This echoes the intrinsic antiviral activities of PML in the context of viral infections. PML NBs can, for example, mediate the epigenetic silencing of viral genomes, entrap newly assembled viral capsids, or interfere with early viral events after relocalization in the cytoplasm (reviewed in reference 55). In the case of *Listeria* infection, we demonstrated that host plasma membrane perforation by LLO triggers PML oxidation, multimerization, and association with the nuclear matrix, in striking similarity to the results of arsenic trioxide exposure. However, in contrast to As_2_O_3_-treated cells, where Ubc9 recruitment into NBs yields a massive PML hyper-SUMOylation (9, 11), PML NBs are paradoxically de-SUMOylated in LLO-treated cells, because of concomitant Ubc9 degradation and, possibly, also recruitment of SUMO proteases. These events generate an unprecedented situation compared to several viral infections that induce PML degradation and/or PML NB disruption to counteract PML antiviral activity (16, 17).

Strikingly, we observed that PML does not control infection by *Salmonella* Typhimurium, even though this pathogen was also shown to induce a decrease in the Ubc9 level (25). Further characterization of the putative PML-regulated genes induced during *Salmonella* infection or the effect of *Salmonella* on PML SUMOylation/multimerization will be required to fully understand the differences in PML-dependent sensitivity between *Listeria* and *Salmonella*. Interestingly, *Shigella flexneri*, in addition to its effect on the Ubc9 level, was shown to induce a two-fold increase in the number of PML NBs in HeLa cells, which was not observed during incubation with a non-invasive avirulent strain (22). The putative role of PML in *Shigella* infection, however, remains unknown.

In conclusion, *Listeria* LLO defines a novel means, i.e., PML de-SUMOylation, through which pathogens unexpectedly activate PML-enforced restriction of their replication. We propose that PML acts as a sensor for bacteria that produce pore-forming toxins, illustrating the concept, initially proposed for viruses but now extended to bacteria, that intranuclear bodies play critical roles in responses against invading pathogens.

We previously demonstrated that the global de-SUMOylation triggered by LLO is actually beneficial for *Listeria* infection (20). The case of PML, whose de-SUMOylation counteracts bacterial infection, illustrates how SUMO alterations of some host proteins can also constitute danger signals for the cells, leading to a response aimed to limit infection. Our data thus highlight the fine balance between toxin-induced alterations of host cell functions that are beneficial for infection and toxin damages sensed by host cells leading to antibacterial responses.

## MATERIALS AND METHODS

### Plasmids.

The cDNAs encoding hCMV iE1^1–328^ and iE1^1–289^ fused to an N-terminal FLAG tag were obtained by gene synthesis (Integrated DNA Technologies, Inc.) and then cloned into the pCDNA.3 vector (Invitrogen) (pCDNA3-FLAG-hCMV iE1^1–382^ [BUG 3779; Bacteria-Cell Interactions laboratory's bacteria collection] and pCDNA3-FLAG-hCMV iE1^1–289^ [BUG 3780]). The plasmids encoding YFP-CBD, a yellow fluorescent protein (YFP) chimera protein of the cell wall binding domain (CBD) from the *Listeria* phage endolysin Ply118 (BUG 2305; kind gift from J. Swanson, University of Michigan Medical School, Ann Arbor, MI, USA), and Sp100 (pSG5-Sp100^WT^ [BUG 4134] and its derivative pSG5-Sp100^K297R^ [BUG 4135]) have been described previously (11, 57).

### Cell culture and transfections.

Mouse embryo fibroblasts (MEFs) derived from *pml*^+/+^ or *pml*^−/−^ mice and immortalized with a plasmid expressing simian virus 40 (SV40) large T antigen and CHO cells stably expressing His_6_-tagged human PML isoform III (CHO-PML) have been described previously (30). These cells were cultivated in Dulbecco modified Eagle medium (DMEM)-GlutaMAX (Invitrogen) supplemented with 10% fetal calf serum (FCS). CHO-PML cells were additionally cultivated with 1 mg/ml hygromycin B (Invitrogen) to maintain the expression of the human PML isoform III (PML-III). HeLa cells (CCL-2 from ATCC [American Type Culture Collection]) were cultivated in minimal essential medium (MEM)-GlutaMAX (Invitrogen) supplemented with 10% FCS, MEM nonessential amino acids (Invitrogen), and 1 mM sodium pyruvate (Invitrogen).

For transfections, HeLa cells and MEFs were seeded at a density of 1.25 × 10^5^ cells per 400-mm^2^ well. The next day, cells were transfected with 1.5 μg of DNA using Lipofectamine LTX reagents (Invitrogen) for 24 h.

### Bacterial strains.

The strains used in this study were *Listeria monocytogenes* strain EGD (BUG 600), an *L. monocytogenes* EGD Δ*hly* mutant (BUG 3650 [58]), *L. monocytogenes* EGDe.PrfA* (BUG 3057), and *Salmonella* Typhimurium strain SR-11 (BUG 3044; kind gift of F. Norel and V. Robbe-Saule, Institut Pasteur, Paris, France). *Listeria* strains were grown in brain heart infusion (BHI) broth or agar plates (BD Difco), whereas *Salmonella* Typhimurium was grown in Luria-Bertani (LB) broth or agar plates (BD Difco).

### Bacterial infections.

For *in vivo* infection, procedures were performed in accordance with protocols approved by the Animal Experimentation Ethics Committee of the Institut Pasteur (permit number 03-49), applying the guidelines of the European Commission for the handling of laboratory animals, Directive 2010/63/EU. The protocols were approved by the veterinary staff of the Institut Pasteur animal facility and were performed in compliance with NIH Animal Welfare Assurance number A5476-1, issued on 31 July 2012. Amounts of 5 × 10^5^ *Listeria* or 1 × 10^5^ *Salmonella* bacteria were injected intravenously into *pml*^+/+^ or *pml*^−/−^ mice (described in reference 59), and the CFUs per organ were enumerated at 72 h postinfection.

For *in vitro* infections, MEFs and CHO-PML cells were seeded, respectively, at a density of 2.5 × 10^5^ or 1.25 × 10^5^ cells per 400-mm^2^ well the day before infection. For transfected HeLa cells, infections were performed 24 h after transfection. Bacteria were cultured overnight, subcultured 1:20 in BHI or LB medium at 37°C until reaching an optical density at 600 nm (OD_600_) of 1.0 for *Listeria* or 1.5 for *Salmonella*, and washed twice in phosphate-buffered saline (PBS). MEFs or CHO-PML or HeLa cells were serum starved for 1 h before the addition of bacteria. Bacteria were added to cells at a multiplicity of infection (MOI) of 50 for *Listeria* or 2 for *Salmonella*. After 1 h of infection, cells were washed and incubated with fresh medium supplemented with 10% FCS and 50 μg/ml gentamicin (Euromedex) to kill extracellular bacteria. For immunoblot analysis, infected cells were lysed 5 h after the beginning of infection with Laemmli buffer (0.125 M Tris, pH 6.8, 4% SDS, 20% glycerol, 100 mM dithiothreitol [DTT], 0.0025% bromophenol blue). To quantify intracellular bacteria, infected cells were lysed 1.5, 5, or 24 h after the beginning of infection with PBS–0.2% Triton X-100 (Sigma), and the number of viable intracellular bacteria released from the cells was assessed by plating on BHI or LB agar plates as previously described (60).

### Analysis of gene expression from infected cells.

RNAs from MEFs infected or not for 24 h with wild-type or Δ*hly Listeria* (EGD strain) were extracted using miRNeasy minikits (Qiagen). Four hundred nanograms of RNA was reverse transcribed using RT^2^ first strand kits (Qiagen). Real-time PCRs were then performed using a CFX384 real-time PCR detection system (Bio-Rad), RT^2^ SYBR green master mix, and RT^2^ Profiler mouse antibacterial PCR arrays (Qiagen). Gene expression was then normalized using the expression levels of 5 reference genes (*actb*, *b2m*, *gapdh*, *gusb*, and *hsp90a1*).

### Analysis of cytokine secretion from infected cells.

Five hundred microliters of supernatants from MEFs infected or not for 24 h with wild-type or Δ*hly Listeria* (EGD strain) were collected, centrifuged for 10 min at 13,000 × *g* to remove cell remnants, and used to probe Proteome Profiler mouse XL cytokine arrays (R&D Systems), following the manufacturer’s instructions. Cytokine levels were quantified using the G:Box gel documentation system and the associated GeneTools software (Syngene).

### Statistical analysis of gene expression and cytokine secretion.

Both gene expression and cytokine secretion data were analyzed using R version 3.3.0 (61) and the limma Bioconductor package version 3.28.2 (62). The replicate effect was included in the linear models as a blocking factor alongside the variables of interest. Raw *P* values were adjusted for multiple testing according to the Benjamini and Hochberg (BH) procedure (63), and features with an adjusted *P* value lower than 0.05 were considered differentially expressed.

### Pore-forming toxins and arsenic treatment.

For treatments with pore-forming toxins or arsenic trioxide (As_2_O_3_, single-element standard solution, 1,000 mg/liter stock solution; Sigma-Aldrich), MEFs and CHO-PML cells were seeded, respectively, at a density of 2.5 × 10^5^ or 1.25 × 10^5^ cells per 400-mm^2^ well the day before treatment. For His pulldown of His_6_-tagged PML, CHO-PML cells were seeded at a density of 2.5 × 10^6^ cells in 75-cm^2^ flasks the day before treatment. Cells were serum starved for 2 h before treatment.

Wild-type LLO (LLO^WT^) and LLO^W492A^ proteins were purified as previously described (64). Purified PFO and PLY were kindly provided by T. Mitchell (University of Glasgow, Glasgow, Scotland, UK). Purified pore-forming toxins were added directly to the culture medium as indicated in the text. PFO and PLY were used at the same hemolytic titre as LLO.

Arsenic trioxide was added to the culture medium at a final concentration of 10 μM for 1 h. For proteasome inhibition, CHO-PML cells were pretreated with 10 μM MG132 (Z-Leu-Leu-Leu-al; Sigma-Aldrich) or dimethyl sulfoxide (DMSO) for 5 h, washed, and then incubated with LLO or As_2_O_3_. *N*-Ethyl-maleimide (NEM; Sigma-Aldrich) was added to the culture medium for 1 h, and cells were then washed and incubated with LLO or As_2_O_3_. After treatment, cells were lysed directly in Laemmli buffer. For analysis under nonreducing conditions, cells were lysed in Laemmli buffer without DTT.

### His pulldown assays.

His_6_-tagged PML was isolated from CHO-PML cells lysed with lysis buffer (6 M guanidium HCl, 10 mM Tris, 100 mM sodium phosphate buffer [pH 8.0], 5 mM β-mercaptoethanol, 1 mM imidazole). The cell lysates were sonicated and centrifuged for 15 min at 16,000 × *g*, and the corresponding supernatants were incubated overnight at 4°C with 250 μl of packed Ni-nitrilotriacetic acid (NTA) agarose beads (Qiagen) prewashed in lysis buffer. After incubation, the beads were washed once in lysis buffer, once in wash buffer, pH 8.0 (8 M urea, 10 mM Tris, 100 mM sodium phosphate buffer [pH 8.0], 0.1% Triton X-100, 5 mM β-mercaptoethanol), and three times in wash buffer, pH 6.3 (8 M urea, 10 mM Tris, 100 mM sodium phosphate buffer [pH 6.3], 0.1% Triton X-100, 5 mM β-mercaptoethanol, 10 mM imidazole). His_6_-tagged PML proteins were then eluted from the beads using elution buffer (200 mM imidazole, 5% SDS, 150 mM Tris-HCl [pH 6.7], 30% glycerol, 720 mM β-mercaptoethanol, 0.0025% bromophenol blue).

### Flow cytometry analysis.

CHO cells were treated with 0.3 nM LLO for 2 min, washed, and then labeled with 1 μM CM-H_2_DCFDA (chloromethyl derivative of 2′,7′-dichlorodihydrofluorescein diacetate; Life Technologies, Inc.) for 20 min. Cells were then detached using Versene solution and analyzed with a FACSCalibur flow cytometer (Becton, Dickinson).

### *In situ* nuclear matrix preparation.

Nuclear matrices were prepared as described previously (11). CHO-PML cells were seeded at a density of 3 × 10^5^ cells on 10-cm^2^ coverslips or 2.5 × 10^6^ cells in 80-cm^2^ plates the day before treatment. After LLO or As_2_O_3_ treatment, cells were fixed for 15 min at 4°C in Kern matrix buffer (KMB) (10 mM MES [morpholineethanesulfonic acid], pH 6.2, 10 mM NaCl, 1.5 mM MgCl_2_, protease inhibitors [complete protease inhibitor cocktail tablets; Roche], 10% glycerol) and then washed twice with KMB containing 1% NP-40. After three additional washes with KMB, cells were incubated with 50 μg/ml RNase A and 0.3 U/ml micrococcal nuclease for 30 min at 25°C. Cells were then washed three times with KMB containing 2 M NaCl for 15 min at 4°C and three times with KMB. Nuclear matrices were then resuspended in Laemmli buffer for immunoblot analysis or fixed in PBS–4% paraformaldehyde (PFA) for 10 min, followed by 100% methanol for 5 min, for immunofluorescence analysis.

### Western blot analysis.

Cells lysed in Laemmli buffer, proteins eluted from His pulldown assays, and nuclear matrix preparations were resolved by SDS-polyacrylamide gel electrophoresis. Proteins were then transferred to polyvinylidene difluoride (PVDF) membranes and detected after incubation with specific antibodies with Pierce enhanced chemiluminescence (ECL) 2 Western blotting substrate (Fisher Scientific). The primary antibodies used for immunoblot analysis are described in Table S4 in the supplemental material. Rabbit polyclonal antibodies against LLO (R176), SUMO1 (R204), and SUMO3 (R205 and R206) were obtained in-house by immunizing rabbits with recombinant proteins produced in *Escherichia coli*, followed by affinity purification of the immune serum. Antibodies against human PML were obtained in-house from chicken eggs immunized with glutathione *S*-transferase (GST)–PML-III fusion protein produced in *Escherichia coli* (34). Antibodies against human Sp100A were obtained in-house by immunizing rabbits with recombinant full-length hSp100A (11). Anti-mouse and anti-rabbit horseradish peroxidase (HRP)-conjugated antibodies (AbCys) were used as secondary antibodies. All immunoblots displayed in the figures are representative of at least two independent experiments.

10.1128/mBio.02179-16.9 Table S4  Primary antibody information. Download Table S4, PDF file, 0.1 MB. Copyright © 2017 Ribet et al. 2017 Ribet et al. This content is distributed under the terms of the Creative Commons Attribution 4.0 International license.

### Immunofluorescence analysis.

Cells on coverslips were incubated after methanol fixation with primary and then secondary antibodies in PBS–1% BSA, and mounted in Fluoromount (Interchim). The antibodies used for immunofluorescence analysis are described in Table S4 in the supplemental material. Mouse monoclonal antibody against human PML (2′C7) was obtained from murine hybridoma in-house using purified PML-III–MBP fusion protein. Alexa Fluor 488- or 546-labeled anti-mouse and anti-rabbit antibodies (Molecular Probes), Texas red-labeled anti-goat antibodies, and fluorescein isothiocyanate (FITC)-conjugated anti-mouse antibodies (Jackson Immunology) were used as secondary antibodies. All images displayed in the figures are representative fields from at least two independent experiments.
