# Supplementary material for: Promyelocytic Leukemia Protein (PML) Controls Listeria monocytogenes Infection
Source: mBio. 2017 Jan 10;8(1):e02179-16. doi: 10.1128/mBio.02179-16 (PMC5225316; doi:10.1128/mBio.02179-16)
Supplement: FIG S1 [file mbo001163144sf1.pdf]

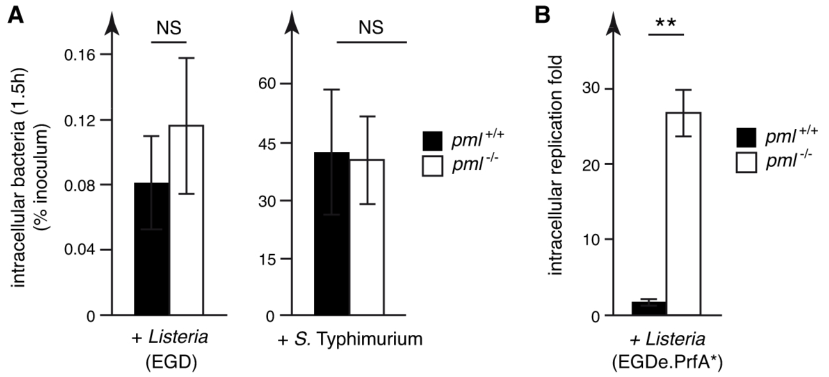

Figure S1 : PML restricts bacteria intracellular replication but not bacterial entry in MEFs. MEFs derived from *pml*$^{+/+}$ or *pml*$^{-/-}$ mice were infected by *L. monocytogenes* or *S.* Typhimurium. (A) Histograms correspond to the numbers of intracellular bacteria recovered after 1.5 h of infection, expressed as the percentage of the inoculum initially used for infection (mean results ± SEM from 6 to 8 independent experiments; NS, not significant; unpaired two-tailed Student's *t* test). No significant differences were observed in bacterial numbers, suggesting that the internalization efficiencies of *Listeria* and *Salmonella* are similar in both *pml*$^{+/+}$ and *pml*$^{-/-}$ MEFs. (B) Histograms correspond to fold changes in bacterial intracellular replication, expressed as the ratio of intracellular bacteria at 24h versus 1.5h of infection (mean results ± SD from 3 independent experiments; **, $P < 0.01$; unpaired two-tailed Student's *t* test). A significant increase in the replication efficiency is observed in *pml*$^{-/-}$ MEFs compared to that of *pml*$^{+/+}$ MEFs, indicating that PML restricts the intracellular replication of *Listeria* strain EGDe.PrfA*.
