# Supplementary material for: Promyelocytic Leukemia Protein (PML) Controls Listeria monocytogenes Infection
Source: mBio. 2017 Jan 10;8(1):e02179-16. doi: 10.1128/mBio.02179-16 (PMC5225316; doi:10.1128/mBio.02179-16)
Supplement: FIG S2 [file mbo001163144sf2.pdf]

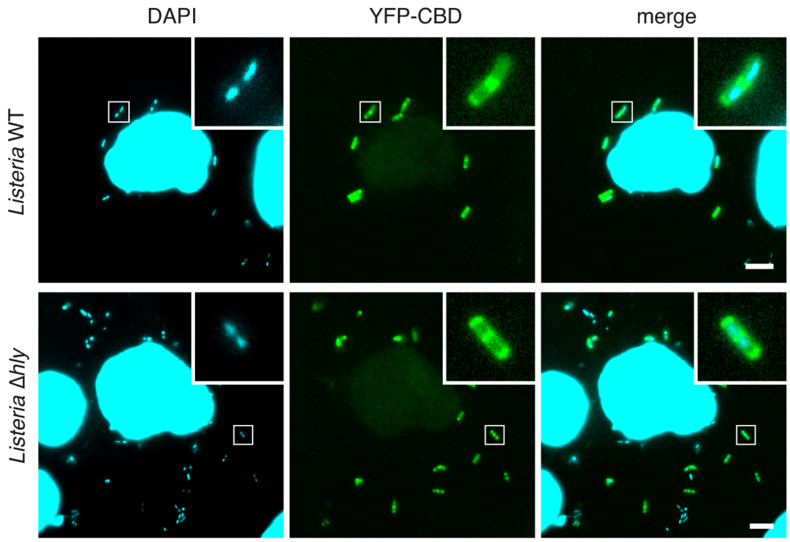

Figure S2 : Detection of *Listeria* vacuolar escape in MEFs. Immunofluorescence analysis of *pml*$^{+/+}$ MEFs transfected with an expression vector for YFP-CBD, a YFP chimera protein of the cell-wall binding domain from the *Listeria* phage endolysin Ply118 (R. Henry, L. Shaughnessy, M.J. Loessner, C. Alberti-Segui, D.E. Higgins and J.A. Swanson, Cell Microbiol 8:107-119, 2006, doi:10.1111/j.1462-5822.2005.00604.x). MEFs were infected 24 h after transfection with wild-type or Δ*hly Listeria monocytogenes*. After 1 h of infection, cells were washed and incubated for an additional 4 h in culture medium supplemented with gentamicin to kill extracellular bacteria; intracellular bacteria remain unaffected by this antibiotic. Cells were then fixed, and bacterial and cellular DNAs were stained with 4',6-diamidino-2-phenylindole (DAPI) (scale bar, 5 µm). The YFP-CBD protein, synthesized in the host cell cytoplasm, recognizes and binds only to bacteria that escaped from the internalization vacuole. Here, CBD-positive Δ*hly Listeria* cells can be detected, indicating that at least a fraction of these bacteria can escape the internalization vacuole in this murine cell line and reach the host cell cytosol, as already observed for several human cell lines (M.A. Hamon, D. Ribet, F. Stavru and P. Cossart, Trends Microbiol 20:360-368, 2012, doi:10.1016/j.tim.2012.04.006).
