# Supplementary material for: Promyelocytic Leukemia Protein (PML) Controls Listeria monocytogenes Infection
Source: mBio. 2017 Jan 10;8(1):e02179-16. doi: 10.1128/mBio.02179-16 (PMC5225316; doi:10.1128/mBio.02179-16)
Supplement: FIG S3 [file mbo001163144sf3.pdf]

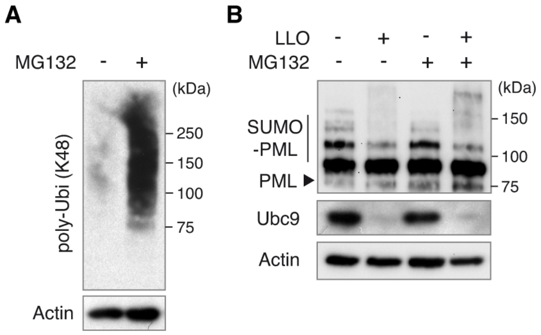

Figure S3 : LLO induces PML de-SUMOylation rather than degradation. (A) Immunoblot analysis using anti-K48-linked polyubiquitin and anti-actin antibodies of whole-cell lysates from CHO-PML cells preincubated with 10 µM MG132 for 5 h. The level of proteins conjugated to K48-polyubiquitin chains increased after MG132 treatment, thus validating proteasome inhibition under these conditions. (B) Immunoblot analysis using anti-PML, anti-Ubc9 and anti-actin antibodies of whole-cell lysates from CHO-PML cells preincubated with 10 µM MG132 for 5 h and then treated with 1 nM LLO for 20 min. Pretreatment with MG132 does not block loss of PML SUMOylated forms, suggesting that LLO triggers PML de-SUMOylation rather than degradation.
