# Supplementary material for: Promyelocytic Leukemia Protein (PML) Controls Listeria monocytogenes Infection
Source: mBio. 2017 Jan 10;8(1):e02179-16. doi: 10.1128/mBio.02179-16 (PMC5225316; doi:10.1128/mBio.02179-16)
Supplement: FIG S4 [file mbo001163144sf4.pdf]

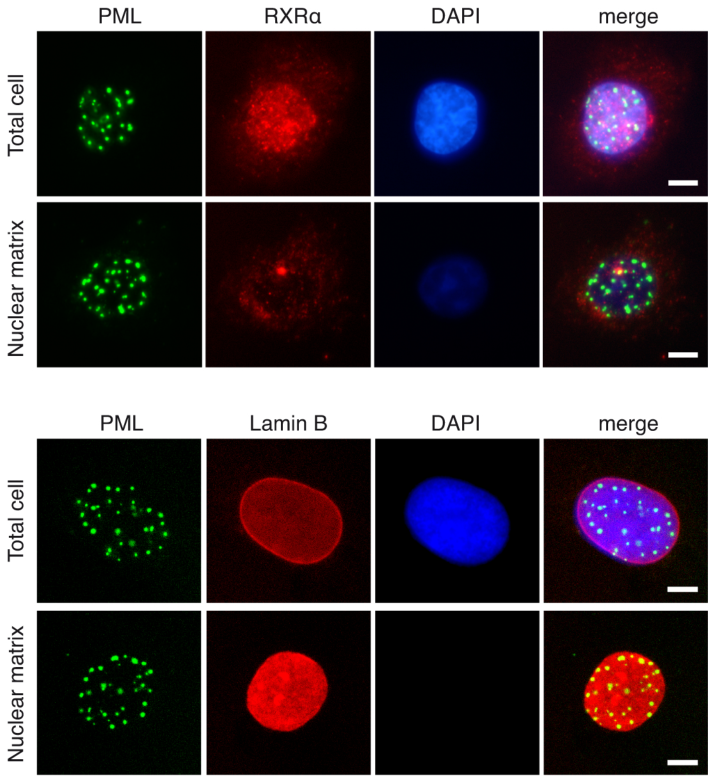

Figure S4 : Control of *in situ* nuclear matrix preparations. Immunofluorescence analysis of total nuclei from CHO-PML cells or nuclear matrix preparations using DAPI, anti-PML, anti-lamin B and anti-RXRα antibodies. As expected, after the high salt extraction and nuclease treatment used for nuclear matrix preparation, the nuclear soluble RXRα factor and DNA were removed, whereas lamin B and NB associated-PML, which belong to the nuclear matrix fraction, are still present. Scale bar, 5 µm.
