# Supplementary material for: Promyelocytic Leukemia Protein (PML) Controls Listeria monocytogenes Infection
Source: mBio. 2017 Jan 10;8(1):e02179-16. doi: 10.1128/mBio.02179-16 (PMC5225316; doi:10.1128/mBio.02179-16)
Supplement: FIG S5 [file mbo001163144sf5.pdf]

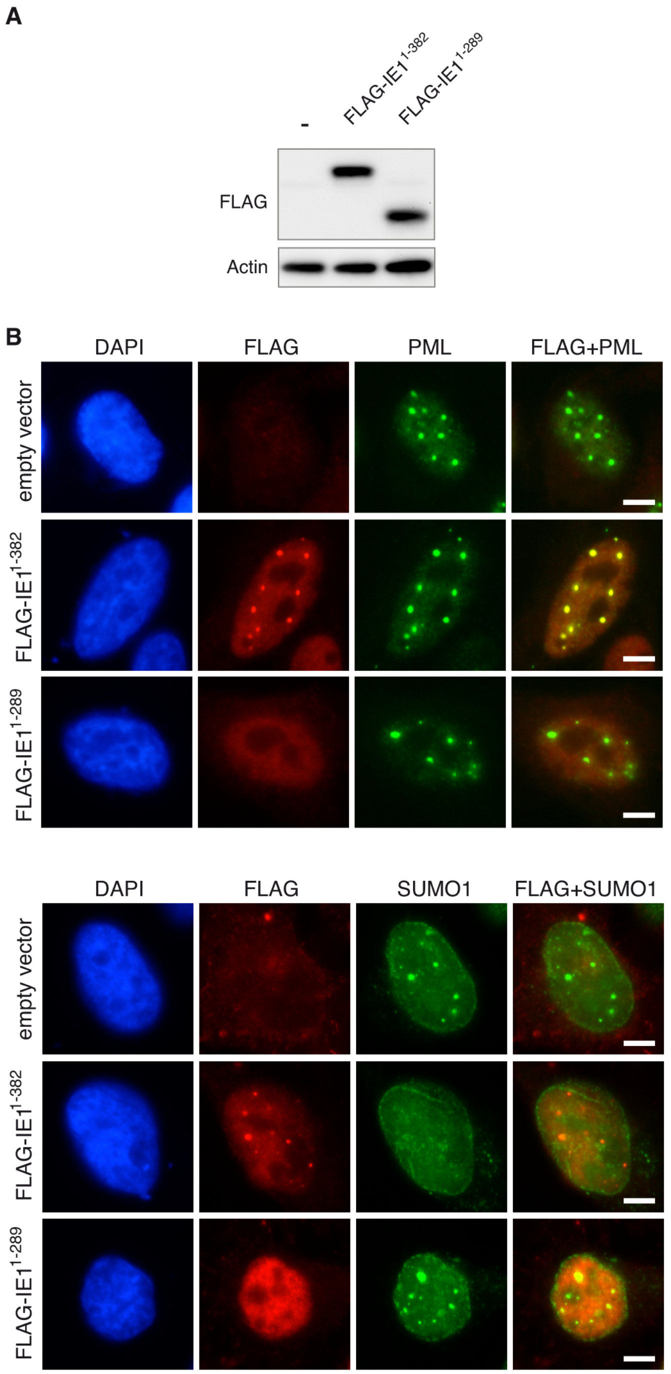

Figure S5 : hCMV iE1$^{1\text{-}382}$ protein binds to PML and induces its de-SUMOylation. (A) Immunoblot analysis using anti-FLAG and anti-actin antibodies of HeLa cells transfected with pCDNA.3 empty vector or expression vectors for FLAG-iE1$^{1\text{-}382}$ or iE1$^{1\text{-}289}$. (B) Immunofluorescence analysis of nuclei from transfected HeLa cells stained with DAPI, anti-FLAG, anti-PML and anti-SUMO1 antibodies. The iE1$^{1\text{-}382}$ protein displays a nuclear localization, binds to PML, and induces its de-SUMOylation. The iE1$^{1\text{-}289}$ protein also has a nuclear localization but does not bind or de-SUMOylate PML. Scale bar, 5 µm.
