# Supplementary material for: Promyelocytic Leukemia Protein (PML) Controls Listeria monocytogenes Infection
Source: mBio. 2017 Jan 10;8(1):e02179-16. doi: 10.1128/mBio.02179-16 (PMC5225316; doi:10.1128/mBio.02179-16)
Supplement: Table S4 [file mbo001163144st4.pdf]

Supplementary Table S4 : Primary antibody information.

Targeted Protein	Assay (dilution)	Species	Source	Reference
Actin	WB (1:10,000)	Mouse	Sigma-Aldrich	R5441
Ubc9	WB (1:1,000)	Mouse	BD Biosciences	610748
K48-linked polyubiquitin	WB (1:1,000)	Rabbit	Cell Signaling Technology	D9D5; #8081
LLO	WB (1:20,000)	Rabbit	Home-made	R176
PML	WB (1:2,000)	Chicken	Home-made	
PML	IF (1:500)	Mouse	Home-made	2'C7
Sp100	WB (1:2,000)	Rabbit	Home-made	
SUMO1	WB (1:1,000)	Rabbit	Home-made	R204 (p)
SUMO1	IF (1:100)	Rabbit	Cell Signaling Technology	#4930
SUMO3	WB (1:5,000)	Rabbit	Home-made	R205 (is2)
SUMO3	IF (1:500)	Rabbit	Home-made	R206 (p)
Lamin B	IF (1:200)<br>WB (1:1,000)	Goat	Santa Cruz Biotechnology	M-20; sc-6217
RXRα	IF (1:200)	Rabbit	Santa Cruz Biotechnology	D-20; sc-553
FLAG	WB (1:1,000)<br>IF (1:200)	Mouse	Sigma-Aldrich	M2; F3165
FLAG	IF (1:200)	Rabbit	Sigma-Aldrich	F7425
